# High-fidelity simulation in airway management training: results from the AIR-MASTER-SIM randomised study

**DOI:** 10.3389/fmed.2026.1829702

**Published:** 2026-06-01

**Authors:** Javier Puerma-Jiménez, José Miguel Pérez-Villares, Pedro Domínguez-Guzmán, Gerardo Gómez-Moreno, Francisco Manuel Parrilla-Ruiz, Ana Carrasco-Cáliz, Pedro Fernández-Florido, Antonio Cárdenas-Cruz

**Affiliations:** 1 Intensive Care Department, Hospital Universitario Virgen de las Nieves, Granada, Spain; 2 Research Group CTS-609, Granada, Spain; 3 Research Group TEC 23, Instituto de Investigación Biosanitaria de Granada (IBS Granada)-ISCIII, Granada, Spain; 4 Department of Medicine, Faculty of Medicine, University of Granada, Granada, Spain; 5 Department of Stomatology, Faculty of Dentistry, University of Granada, Granada, Spain; 6 Research Group CTS-654, Granada, Spain; 7 Emergency Department, Hospital Universitario Clínico San Cecilio, Granada, Spain; 8 8Intensive Care Service, Hospital Universitario, Virgen Macarena, Sevilla, Spain

**Keywords:** airway management, direct laryngoscopy, endotracheal intubation, medical education, medical students, simulation-based training, video laryngoscopy

## Abstract

**Introduction:**

Airway management is a key competency in emergency medicine and critical care, and effective training in endotracheal intubation is essential during undergraduate medical education. Direct laryngoscopy has traditionally been considered the standard technique for airway access, although it can present technical difficulties for inexperienced operators. Videolaryngoscopy provides indirect visualisation of the glottis and may facilitate airway management during the early stages of training. This study compared the acquisition of airway management skills among medical students trained using direct laryngoscopy or videolaryngoscopy in a simulation-based educational environment.

**Methods:**

A prospective, randomised, comparative simulation-based study was conducted among fourth-year medical students at the University of Granada (Spain). Participants were assigned to direct laryngoscopy or videolaryngoscopy training. Training and assessment were conducted in a high-fidelity clinical simulation environment using standardised scenarios designed to assess cognitive, procedural and attitudinal competencies related to airway management. The main outcomes included the intubation success rate, the time required to achieve airway access, the number of attempts required to achieve successful intubation and the occurrence of procedure-related complications. Perceived stress and perceived control of procedure time during the simulation scenario were also recorded.

**Results:**

A total of 305 medical students participated in the study, including 152 in the direct laryngoscopy group and 153 in the videolaryngoscopy group. Airway access was achieved in 98.0% of students trained with videolaryngoscopy compared to 7.9% of those trained with direct laryngoscopy. Videolaryngoscopy was associated with fewer attempts required to achieve successful intubation, shorter procedure time and fewer procedure-related complications. Trainees trained with videolaryngoscopy also reported greater perceived control of procedure time and lower levels of stress during the simulation scenario.

**Conclusion:**

Videolaryngoscopy facilitated the acquisition of airway management skills during the early stages of medical training. The improved view of airway structures and the possibility of shared visualisation between instructors and trainees may partly explain the better procedural performance observed in the simulation setting.

## 1 Introduction

Airway management represents one of the most critical competencies in emergency and critical care medicine. Securing the airway by endotracheal intubation is often necessary in life-threatening situations such as acute respiratory failure, trauma or cardiac arrest, where rapid and effective airway management is essential to maintain oxygenation and ventilation. Failure to perform endotracheal intubation correctly can lead to serious complications such as hypoxia, lung injury, neurological damage or even death (1, 2). Airway management is an important competency in emergency and critical care contexts. However, endotracheal intubation should be understood as a context-dependent procedural skill rather than a universal core competency expected of all medical graduates. Undergraduate medical education should ensure that students understand airway anatomy, physiology, oxygenation, ventilation and the principles of airway assessment, whilst procedural airway instrumentation requires specific training, repeated supervised practice and progressive exposure. Within this framework, simulation-based training provides a safe environment to introduce novice learners to airway management techniques and to assess early procedural learning before any clinical exposure. Therefore, proper training in airway management is widely recognised as a fundamental component of undergraduate medical education and an essential skill for future physicians to acquire before entering clinical practice. For decades, direct laryngoscopy has been considered the gold standard technique for tracheal intubation. This technique is based on direct visualisation of the glottic structures using a laryngoscopic blade, which requires proper alignment of the oral, pharyngeal and laryngeal axes to achieve adequate visualisation of the vocal cords. Despite its widespread use in clinical practice, direct laryngoscopy presents several challenges, especially for inexperienced operators. Difficult airway anatomy, limited visualisation of airway structures and the need for extensive technical experience may increase the learning curve and reduce the likelihood of achieving successful intubation on the first attempt (2–4). In addition, repeated intubation attempts have been associated with an increased risk of complications and adverse clinical events in emergency situations (5, 6).

In recent years, video laryngoscopy has emerged as an important technological advance in airway management. Videolaryngoscopes incorporate a miniature camera at the distal end of the blade, allowing indirect visualisation of the glottis through a video monitor. This approach provides a wider and often clearer view of airway structures, which facilitates the identification of anatomical landmarks and may improve the success rate of intubation procedures. Video laryngoscopy has been shown to be especially useful in patients with anatomically difficult airways or when intubation is performed by less experienced physicians (7–9). In addition, several studies have suggested that videolaryngoscopy may increase first-attempt success rates and reduce airway-related complications during emergency intubation procedures (5–8). Beyond its clinical advantages, videolaryngoscopy also offers important opportunities in the field of medical education. Because the view of the airway obtained during videolaryngoscopy can be simultaneously displayed to both instructors and trainees, this technology facilitates real-time monitoring, immediate feedback and shared visualisation of airway anatomy during training and education. These features can enhance the educational process by allowing instructors to guide trainees more effectively during airway management procedures. In addition, modern videolaryngoscopy systems can incorporate visualisation technologies that facilitate anatomical understanding and procedural guidance, which may contribute to more efficient acquisition of technical skills in airway management (7–9).

Medical education is currently undergoing a transformation driven by the integration of innovative educational technologies. Simulation-based training and advanced visualisation tools are increasingly used to enhance the acquisition of procedural skills in complex clinical scenarios. In this context, airway management training represents an ideal field for the implementation of these technologies, especially in simulation environments that allow trainees to develop technical competencies in a safe and controlled environment before performing procedures on real patients (10, 11). Despite the increasing adoption of video laryngoscopy in clinical practice, its optimal role in undergraduate airway management training remains unclear. Medical schools have traditionally taught direct laryngoscopy as the primary technique for tracheal intubation, reflecting its historical role as the standard approach to airway management. However, the increasing availability of video laryngoscopes raises important questions about whether this technology can provide a more effective learning pathway for novice operators and whether it should be incorporated earlier in the training of medical students. Evidence comparing the educational effectiveness of the two approaches in undergraduate medical training remains limited, and further research is needed to determine which teaching strategy best facilitates the acquisition of airway management skills. Therefore, assessing the impact of these two techniques on the acquisition of airway management competencies is essential to optimise training strategies and improve patient safety in future clinical practice.

The aim of this study was to comparatively analyse the acquisition of airway management skills among medical students trained with two different techniques: direct laryngoscopy and videolaryngoscopy. Specifically, the following outcomes were assessed: the first attempt success rate, the time required to achieve satisfactory airway access during the first attempt, the total number of attempts required to achieve successful intubation and the occurrence of local complications associated with each technique during simulated airway management procedures.

## 2 Materials and methods

### 2.1 Ethics committee approval

This study with human participants was approved by the Research Ethics Committee of the University of Granada (Spain), approval code 4542/CEIH/2024. The study complied with the ethical principles governing research with human participants and with the institutional requirements established for this type of educational research. Participation was entirely voluntary and had no impact on academic grading or course evaluation. All eligible students were informed of the study objectives and procedures prior to enrolment, and participation was contingent upon acceptance of the study conditions and ethical procedures.

### 2.2 Study design

A prospective, randomised, simulation-based comparative educational study was conducted to assess the acquisition of airway management competencies among undergraduate medical students trained by direct laryngoscopy or videolaryngoscopy. The study was conducted at the clinical simulation facilities of the Faculty of Medicine, University of Granada (Spain), during the academic years 2024/2025 and 2025/2026, depending on the timing of selection and institutional scheduling. The study was specifically designed within a controlled simulation environment to allow standardised training and assessment of cognitive, procedural and attitudinal competencies related to critical airway management.

### 2.3 Participants and selection

Eligible participants were fourth year medical students enrolled in the Respiratory Pathology course at the University of Granada (Spain) during the study period. None of the participants had received prior specific training in advanced airway management, direct laryngoscopy, videolaryngoscopy or high-fidelity airway simulation before enrolment. Previous simulation exposure was limited to basic life support educational activities unrelated to advanced airway management. Before enrolment in the study, participants had not received previous specific training in airway instrumentation, either with direct laryngoscopy or videolaryngoscopy. All students started the intervention as novice operators regarding endotracheal intubation. The educational activity was therefore designed to assess initial acquisition of airway management competencies after a standardised simulation-based training session, rather than to certify independent clinical competence. Inclusion criteria were enrolment in the course and voluntary agreement to participate in the study. No specific exclusion criteria were established other than refusal to participate. All potentially eligible students received an explanatory email describing the characteristics of the study and were given the opportunity to resolve any doubts before enrolling. The protocol explicitly stated that non-participation would have no academic consequences, as the study was not integrated into the formal grading of the course and the assessed content did not affect the students’ final grade. Although it was hoped to access the entire target population, a minimum sample size was estimated *a priori* to ensure statistical significance and to take into account possible missing or incomplete participation. According to the study protocol, the estimated minimum sample size was 170 participants. The researchers anticipated that recruitment could exceed this threshold and potentially include the entire eligible cohort, estimated at approximately 330 enrolled students. The sample size estimate presented in the protocol was based on a finite population approach. After recruitment and completion of ethical procedures, participants were assigned by simple randomisation to one of the two study groups: the direct laryngoscopy group and the videolaryngoscopy group. The randomisation sequence was generated using a spreadsheet-based randomisation procedure as described in the study protocol. Students assigned to the direct laryngoscopy group received training and assessment using conventional laryngoscopy, while students assigned to the videolaryngoscopy group underwent the same educational process using videolaryngoscopy. Therefore, the comparative design allowed assessment of differences in competence acquisition between the two techniques in the same simulation-based educational setting.

### 2.4 Educational intervention

The educational intervention was designed to provide standardised training in critical airway management using one of the two assigned airway access techniques. The educational intervention consisted of standardised two-hour sessions delivered to small student cohorts. Each session included a structured theoretical introduction, supervised practical instruction, hands-on procedural training using assigned airway devices, and high-fidelity simulation-based assessment. All participants underwent the same standardised instructional sequence, with identical educational objectives, technical exposure and competency assessment criteria.

The educational programme consisted of standardised high-fidelity simulation-based training sessions specifically designed for novice operators. Training followed an experiential learning framework combining structured briefing, supervised procedural practice and scenario-based airway management exercises under expert instructor guidance. All instructors were experienced critical airway management educators with formal training in simulation-based teaching methodology, and all participants completed the same standardised educational pathway prior to assessment.

The training was delivered by instructors with formal airway management experience. According to the study protocol, all participating instructors had a common training in airway instrumentation, and pre-standardisation sessions were planned in order to define and reinforce all aspects of the teaching process, thus minimising inter-instructor variability. The educational process was based on clinical simulation methodology and aimed to assess the acquisition of competencies in three domains: cognitive competencies, procedural competencies and attitudinal competencies. The cognitive domain included knowledge of difficult airway prediction and assessment tools, including the most commonly used airway assessment tools such as the Mallampati classification, the MACOCHA score, the LEMON airway assessment method and the Cormack–Lehane laryngoscopic classification, as well as knowledge of the technical resources used in critical airway management and an understanding of the advantages and limitations of both direct laryngoscopy and video laryngoscopy. Students were also instructed in the identification of airway difficulty using the POGO (percentage of glottic opening) score. The procedural domain included pre-assessment of the airway, preparation of the necessary equipment, on-site assessment of the airway and successful performance of the assigned technique. The attitudinal domain included scenario control, time management and stress management during the simulation of the performance of the procedure.

### 2.5 Simulation environment and evaluation procedure

The methodological basis of the study was high-fidelity clinical simulation, which was selected because it allowed assessment of the three dimensions of competence under controlled and standardised conditions. All participants were assessed using the same predefined airway management simulation scenario, specifically designed for novice undergraduate training. The simulation model represented an anatomically normal airway without additional difficult airway modifiers. Scenario complexity, technical conditions and cognitive demands were standardised across all participants in order to minimise variability and reduce potential performance bias. Simulation equipment included a GlideScope Titanium® videolaryngoscope (Verathon, Bothell, WA, USA), a conventional Macintosh direct laryngoscope, an Airway Larry® airway management simulator (Laerdal Medical, Stavanger, Norway), a Proview 12 multiparameter monitor (ECONET GmbH, Germany), and an Oxylog 3000 ventilator (Dräger, Lübeck, Germany), together with standard consumable airway management materials. The Airway Larry® simulator (Laerdal Medical) used in this study is a high-fidelity airway training manikin specifically designed for teaching airway management techniques, including bag-mask ventilation, direct laryngoscopy, supraglottic airway device insertion and endotracheal intubation. The simulator realistically reproduces normal upper airway anatomy, including the oral cavity, tongue, epiglottis, vocal cords and proximal trachea, thereby allowing standardised, reproducible technical practice under controlled educational conditions. The simulator was used exclusively in its conventional normal-airway configuration without difficult airway modifiers, which was considered appropriate for the study objective of assessing initial competence acquisition in standard airway management scenarios. The simulator was used in its standardised normal-airway configuration for all participants, without modifications for difficult airway scenarios. All students performed airway management procedures under identical anatomical and environmental conditions, ensuring full scenario homogeneity and minimising bias related to anatomical variability. The educational objective was not to reproduce complex pathological airway situations, but rather to assess the initial acquisition of technical airway management competencies in a controlled, reproducible high-fidelity simulation setting.

All equipment was configured identically for each participant to ensure methodological consistency, reproducibility and standardised assessment conditions across all simulation sessions. The simulation model was maintained under anatomically normal airway conditions for all participants, without difficult airway modifiers, thereby ensuring homogeneous technical exposure and minimising scenario-related variability. This standardised equipment configuration allowed objective evaluation of procedural performance, airway access success, predefined time criteria and procedure-related complications, including excessive dental pressure, traumatic airway manipulation and technical errors, under controlled educational conditions. Once the training process was completed, each participant individually performed the assigned technique in the simulation environment. All participants were assessed using the same predefined high-fidelity airway management simulation scenario, specifically designed to evaluate the initial acquisition of direct laryngoscopy and videolaryngoscopy competencies in novice undergraduate medical students. The scenario simulated an adult patient (65 years old) with acute respiratory failure in a standardised intensive care unit environment using a high-fidelity manikin configured with anatomically normal airway conditions and without difficult airway modifiers. Scenario complexity was maintained at a basic-intermediate level for all participants. The technical objective was successful oropharyngeal intubation, whilst non-technical objectives included teamwork, communication, time management and stress control. All simulation sessions followed an identical structure consisting of structured briefing, role allocation, supervised procedural execution and standardised debriefing using the PEARLS framework. Briefing included scenario objectives, simulator familiarisation, psychological safety principles, assigned responsibilities and performance expectations. Standardised role allocation included team leader, airway operator, assistant and observer. Scenario progression was identical for all participants and included initial assessment, equipment preparation, assigned technique execution, confirmation of tube placement and structured post-scenario debriefing. Objective performance was assessed using predefined standardised criteria, including material preparation, technical execution, time to airway access, first-pass success and procedure-related complications. All participants completed two-hour standardised training sessions without interruption, preceded by preparatory cognitive training through the University of Granada e-learning platform. A total of 15 standardised sessions were conducted, ensuring full methodological consistency across all student cohorts. Scenario design, technical difficulty, cognitive load, emotional environment and supervision were deliberately standardised to minimise bias and maximise reproducibility. To minimise potential methodological bias and enhance internal validity, several predefined control measures were systematically incorporated into the study design. All participants were assessed under identical educational and technical conditions using the same standardised simulation scenario, thereby eliminating variability related to scenario design, airway complexity and environmental factors. Previous specific experience in airway instrumentation was excluded by participant selection criteria, ensuring a homogeneous novice population. Objective predefined performance criteria were consistently applied across all participants to standardise assessment of procedural success, time performance and complication rates. Cognitive load and emotional stressors were controlled through homogeneous scenario conditions, structured briefing and consistent psychological safety protocols. In addition, all educational sessions were supervised by instructors with standardised expertise in critical airway management and simulation-based teaching, reducing inter-instructor variability and reinforcing methodological consistency. Collectively, these bias-control measures were specifically implemented to strengthen reproducibility, minimise confounding variables and ensure that the observed differences between direct laryngoscopy and videolaryngoscopy primarily reflected differences attributable to the educational intervention itself rather than external methodological influences.

Additionally, psychological safety was actively protected throughout all sessions through structured supervision and expert soft-skills support. Standardised briefing and debriefing protocols were applied consistently across all sessions to ensure methodological uniformity, optimise educational consistency and minimise instructor-related variability. During this evaluation phase, predefined cognitive, procedural and attitudinal competencies were recorded, as well as measures of efficacy and local complications, using a standardised data collection form. Participant performance was assessed using a structured competency-based evaluation framework specifically developed for this study, incorporating cognitive, procedural, objective and non-technical domains. Assessment criteria were predefined, standardised and based on transparent operational descriptors to maximise reproducibility and minimise evaluator subjectivity. All evaluators underwent prior calibration and shared standardised expertise in critical airway management and simulation-based education. The data collection sheet shown in the protocol included 14 parameters, including knowledge of airway scales, material preparation, technical success, time taken to perform the technique, local complications, total number of attempts, and control of scenario, time and stress. A GREET-based methodological framework was applied to reinforce reporting transparency, including standardisation of participants, intervention, scenario design, assessment criteria and bias control measures.

### 2.6 Outcome measures and study variables

The main objective of the study was to compare the acquisition of airway management skills between medical students trained by direct laryngoscopy and those trained by videolaryngoscopy. According to the study protocol, the main outcome measures related to technical efficacy included the first attempt success rate, the time required to achieve airway access during the first attempt, the total number of attempts required to achieve successful intubation and the occurrence of local complications associated with the procedure. Competency-related variables were organised into three main domains: cognitive, procedural and attitudinal competencies. The cognitive domain included the students’ knowledge of difficult airway prediction scales, their understanding of the resources and techniques used in critical airway management, their knowledge of *in situ* airway classification systems, and their understanding of the advantages and limitations of both direct laryngoscopy and video laryngoscopy. The procedural domain comprised the practical steps required for airway instrumentation, including pre-assessment of the airway, preparation of the necessary equipment, on-site assessment of the airway and successful execution of the assigned airway management technique. The attitudinal domain included behavioural and situational competencies observed during the simulation scenario, namely control of the clinical situation, time management during the procedure and the ability to maintain adequate stress control while performing the technique. Local complications related to airway instrumentation were also recorded using the simulation devices. For the purposes of this study, “specialised equipment” was operationally defined as the use of advanced airway management devices beyond conventional Macintosh direct laryngoscopy, specifically videolaryngoscopy systems and associated visualisation technologies available during the educational intervention. This included GlideScope® videolaryngoscopy as the primary assigned device, together with exposure to additional advanced videolaryngoscopy platforms for educational familiarisation. In contrast, the direct laryngoscopy group used conventional Macintosh laryngoscopy equipment only. Given that the Airway Larry® simulator does not permit direct anatomical assessment of deep or non-visible tissue injuries, procedure-related complications were operationally defined as observable indirect indicators of potentially harmful technical performance during simulation. In this context, complications included behaviours or procedural events that, in real clinical practice, would be associated with increased risk of patient injury, such as traumatic manipulation, excessive force, repeated failed attempts or inappropriate device handling. Following each simulation session, the manikin was systematically inspected for visible evidence of technical misuse or simulator-detectable injury. Although deep structural injury could not be directly confirmed, this operational framework provided a reproducible and educationally relevant measure of procedural safety and technical risk. These complications included dental injuries, upper airway injuries and lower airway injuries detected during the procedure.

Procedure-related complications were identified through direct structured observation by calibrated expert assessors using predefined objective criteria and standardised checklists. Observable indicators included excessive pressure on dental structures (detected through simulator-incorporated audible alarms), traumatic oropharyngeal manipulation, incorrect device insertion, repeated or forceful laryngoscope movements and excessive procedural attempts. For more severe simulated complications, such as potential distal airway or pulmonary injury, identification was based on objective technical inference derived from unsafe procedural behaviours, including forced advancement or inappropriate tube placement, rather than direct anatomical visualisation. This structured observational framework allowed reproducible assessment of technical safety despite the inherent limitations of simulation-based injury modelling. The use of indirect procedural indicators to assess complication risk is an accepted methodological approach in simulation-based medical education, particularly during early-stage technical skills acquisition. This framework allows procedural safety, technical performance and risk-related behaviours to be systematically evaluated in a controlled educational environment without exposing real patients to unnecessary harm. Accordingly, this approach was considered fully consistent with the educational, ethical and methodological objectives of the present study.

Several measures were implemented in the study protocol to minimise potential sources of bias. The investigators recognised that participating trainees could differ in terms of prior experience, technical ability or prior exposure to simulation-based training. To address this potential variability, the protocol incorporated a standardised preparatory process so that all participants began the training intervention under comparable conditions. All participants were assessed using the same simulation-based methodological framework and the same standardised assessment structure, which ensured consistency in the assessment of competencies across study groups. All instructors participating in the educational intervention shared a similar professional background in airway management and participated in pre-coordination meetings designed to standardise the instructional approach and reduce inter-instructor variability in teaching and assessment. Participation in the study was strictly voluntary and independent of the academic grading system. This measure was intended to minimise coercion and reduce performance bias related to formal course evaluation.

### 2.7 Statistical analysis

Statistical analyses were conducted using SPSS Statistics version 25 (IBM Corp., Armonk, NY, USA). First, a descriptive analysis of the entire sample was performed, including absolute and relative frequencies of categorical variables and measures of central tendency and dispersion (mean, standard deviation, median, interquartile range and range) of quantitative variables. Prior to the inferential analyses, the distribution of quantitative variables was assessed to determine whether parametric or non-parametric statistical tests were appropriate. Unpaired dichotomous categorical variables were analysed using contingency tables together with the chi-square test or Fisher’s exact test. Paired dichotomous qualitative variables were analysed using McNemar’s exact test. Comparisons between quantitative variables and dichotomous qualitative variables were performed using Student’s *t*-test for normally distributed data or the Mann–Whitney *U*-test for non-parametric data. Comparisons involving more than two categories were performed using analysis of variance (ANOVA) for parametric distributions or the Kruskal–Wallis test for non-parametric distributions, followed by appropriate *post hoc* analyses when necessary. Associations between quantitative variables were explored using Pearson or Spearman correlation coefficients depending on the distribution of the data. Statistical significance was set at *p* < 0.05.

## 3 Results

### 3.1 Distribution of participants and descriptive results

A total of 305 medical students participated in the simulation-based airway management training programme. Of these, 152 students were assigned to the direct laryngoscopy (DL) group and 153 to the videolaryngoscopy (VL) group. The distribution of the main study variables is shown in Table 1. Baseline cognitive variables related to airway assessment showed no variability between groups. Specifically, Mallampati rating, Cormack–Lehane rating, airway assessment, resource availability and weak/strong airway rating showed identical values in both groups (100%), which prevented statistical comparison. Several procedural and attitudinal variables differed between the groups. The intubation success rate was significantly higher in the VL group. Successful airway access was achieved in 150 students (98.0%) in the VL group compared to 12 students (7.9%) in the DL group. In contrast, failures occurred in 140 students (92.1%) in the DL group and in 3 students (2.0%) in the VL group. The number of attempts required to achieve successful intubation also differed substantially between the groups. In the VL group, 113 students (73.9%) achieved successful intubation on the first attempt, while only 1 student (0.7%) in the DL group achieved success on the first attempt. In contrast, 140 students (92.1%) in the DL group required three attempts, compared to 7 students (4.6%) in the VL group. Procedure-related complications were more frequent in the DL group. Complications occurred in 41 students (27.0%) in the DL group compared to 5 students (3.3%) in the VL group. Successful completion of the procedure within the predefined time interval also differed between the groups. In the VL group, 147 participants (96.1%) achieved satisfactory airway access within the time limit, while this occurred in 11 participants (7.2%) in the DL group. Attitudinal variables related to the simulation scenario also differed between the groups. Participants who trained with videolaryngoscopy showed greater perceived control over procedure time and lower levels of perceived stress than those who trained with direct laryngoscopy. Adequate time control was reported by 121 students (79.1%) in the VL group and 45 students (29.6%) in the DL group. Similarly, 117 students (76.5%) in the VL group and 10 students (6.6%) in the DL group reported stress control. The use of specialised material was also more frequent in the VL group, with 152 students (99.3%) reporting the use of specific material, compared to 139 students (91.4%) in the DL group.

**Table 1:** Comparison of frequencies and percentages between direct laryngoscopy and video laryngoscopy.

Variable	Category	Direct laryngoscopy *n* (%)	Video laryngoscopy *n* (%)
Scenario control	No	19 (12.5%)	24 (15.7%)
	Yes	133 (87.5%)	129 (84.3%)
Stress management	No	142 (93.4%)	36 (23.5%)
	Yes	10 (6.6%)	117 (76.5%)
Time control	No	107 (70.4%)	32 (20.9%)
	Yes	45 (29.6%)	121 (79.1%)
Complications	No	111 (73.0%)	148 (96.7%)
	Yes	41 (27.0%)	5 (3.3%)
Cormack–Lehane classification	Yes	152 (100%)	153 (100%)
Success	No	140 (92.1%)	3 (2.0%)
	Yes	12 (7.9%)	150 (98.0%)
Attempts	1	1 (0.7%)	113 (73.9%)
	2	11 (7.2%)	33 (21.6%)
	3	140 (92.1%)	7 (4.6%)
Mallampati classification	Yes	152 (100%)	153 (100%)
Availability of material	No	13 (8.6%)	1 (0.7%)
	Yes	139 (91.4%)	152 (99.3%)
Resources	Yes	152 (100%)	153 (100%)
Success rate achieved	0	140 (92.1%)	3 (2.0%)
	25	0 (0%)	3 (2.0%)
	50	11 (7.2%)	33 (21.6%)
	75	0 (0%)	1 (0.7%)
	100	1 (0.7%)	113 (73.9%)
Duration of procedure	No	141 (92.8%)	6 (3.9%)
	Yes	11 (7.2%)	147 (96.1%)
Airway assessment	Yes	152 (100%)	153 (100%)
Weak/strong airway classification	Yes	152 (100%)	153 (100%)

### 3.2 Inferential analysis of dichotomous results

**Figure 1:**
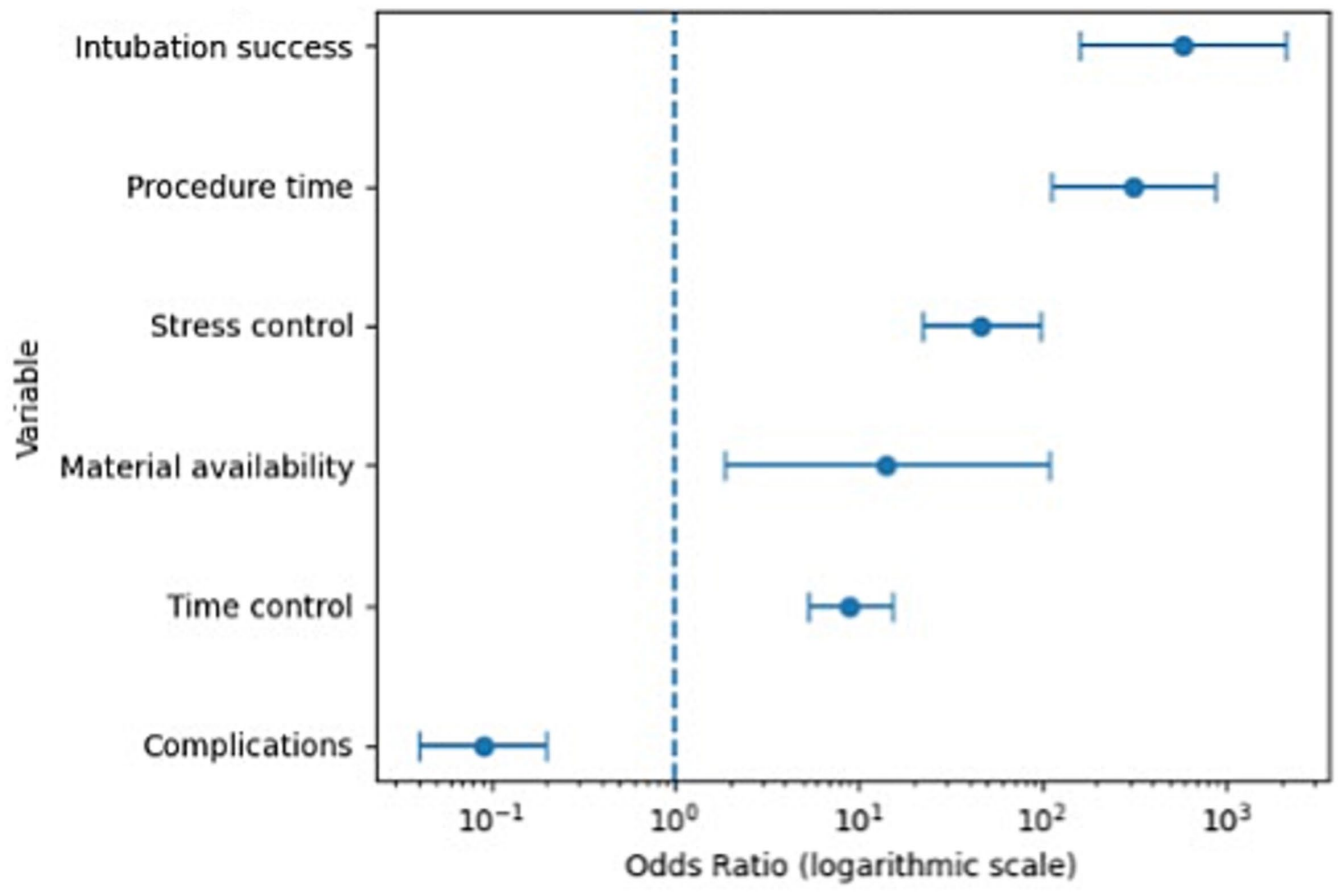
Forest plot of odds ratios (*p* < 0.05). Comparison between direct laryngoscopy (reference) and videolaryngoscopy.

Inferential analysis comparing both airway management techniques is summarised in Table 2. For dichotomous categorical variables, comparisons were performed using Pearson’s chi-square test or Fisher’s exact test, and odds ratios (OR) with 95% confidence intervals (CI) were calculated. Statistically significant differences between groups were observed for several key outcomes. The probability of successful intubation was significantly higher in the VL group, with an OR of 583.33 (95% CI 161.24–2,110.43; *p* < 0.001). VL was also associated with shorter procedure time, with an OR of 314.05 (95% CI 113.12–871.47; *p* < 0.001). The occurrence of complications was significantly lower in the VL group, with an OR of 0.09 (95% CI 0.04–0.20; *p* < 0.001). VL was also significantly associated with greater perceived time control (OR = 8.99, 95% CI 5.33–15.18; *p* < 0.001) and lower stress levels during the simulation scenario (OR = 46.15, 95% CI 21.97–97.07; *p* < 0.001). In addition, the use of specialised equipment was significantly more frequent in the VL group (OR = 14.22, 95% CI: 1.84–109.98; *p* = 0.0025). The associations between airway management technique and the main study outcomes are illustrated in Figure 1.

**Table 2:** Significant associations between airway management technique and study outcomes (odds ratios and 95% confidence intervals).

Variable	*p*-value	Interpretation	OR (VL vs. DL)
Success	0	Highly significant	583.33 (95% CI [161.24–2,110.43]) - success is substantially more likely with video laryngoscopy.
Duration of procedure	0	Highly significant	314.05 (95% CI [113.12–871.47]) - procedure is completed significantly faster with video laryngoscopy.
Complications	0	Highly significant	0.09 (95% CI [0.04–0.20]): video laryngoscopy significantly reduces complications.
Time control	0	Highly significant	8.99 (95% CI [5.33–15.18]): greater perceived time control with video laryngoscopy.
Stress management	0	Highly significant	46.15 (95% CI [21.97–97.07]): video laryngoscopy is associated with significantly lower stress levels.
Material	0.0025	Significant	14.22 (95% CI [1.84–109.98]): video laryngoscopy requires more use of specialised equipment.
Success rate achieved, attempts	0	Highly significant	–

### 3.3 Polytomous variables

For polytomous variables, chi-square tests of independence were performed, and the strength of the association was assessed using Cramer’s *V*. Both the percentage of success achieved and the number of attempts required to achieve successful intubation showed highly significant associations with the airway management technique used (*p* < 0.001). The strength of the association was very high, with a Cramer’s *V* = 0.917 for the percentage of success achieved and a Cramer’s *V* = 0.890 for the number of attempts. These findings demonstrate a strong association between videolaryngoscopy and improved procedural performance.

## 4 Discussion

### 4.1 Main findings

The study showed a marked difference between video laryngoscopy (VL) and direct laryngoscopy (DL) when both techniques were used by medical students with no previous experience in airway management. In the clinical simulation model, the success rate reached 98.0% in the video laryngoscopy group compared with 7.9% in the direct laryngoscopy group, with an odds ratio of 583.33 (95% CI 161.24–2,110.43; *p* < 0.001). In addition, VL was associated with fewer attempts, shorter procedure time, fewer complications and lower levels of perceived stress among operators. These results suggest that the indirect visualisation of the glottis provided by videolaryngoscopy may facilitate airway management during the early stages of training. In this regard, the findings are consistent with a growing body of evidence supporting the benefits of videolaryngoscopy in both clinical and educational settings.

### 4.2 Comparison with previous studies

The findings of this study should be interpreted within the specific framework of undergraduate simulation-based airway education. Among medical students with no previous experience in airway instrumentation, videolaryngoscopy was associated with higher initial intubation success, fewer attempts, shorter procedure times and fewer simulation-detected technical complications than direct laryngoscopy. These findings should not be interpreted as evidence of independent clinical competence, but rather as evidence of improved early procedural performance within a controlled high-fidelity simulation environment.

Comparison with previous literature (Vanderbilt et al., 2014) has therefore been refocused primarily on simulation-based studies, manikin training, novice operators and undergraduate educational settings, consistent with previous educational reviews highlighting the pedagogical role of videolaryngoscopy in simulation-based airway training (12). This distinction is essential because evidence derived from real clinical practice often involves experienced clinicians, variable airway anatomy, physiological instability, secretions, time pressure and patient safety considerations that are not reproduced under educational simulation conditions. Consequently, whilst clinical studies remain valuable for contextualising the broader role of videolaryngoscopy in airway management, they should not serve as direct evidence of educational effectiveness in novice undergraduate learners.

From an educational perspective, videolaryngoscopy may be particularly advantageous during early skill acquisition because airway visualisation can be shared simultaneously between learner and instructor. This pedagogical advantage has been consistently reported in simulation-based educational studies involving undergraduate learners and novice operators, including recent comparative studies (Malito et al., 2023 (13); Öcal, 2025) (14), systematic review evidence (Gunning et al., 2025) (15), and manikin usability analyses (Lee et al., 2024) (16). This shared visualisation facilitates immediate instructor feedback, enhances anatomical orientation and may reduce the technical barriers associated with direct line-of-sight laryngoscopy.

Therefore, the observed differences may reflect not only device-related advantages but also important pedagogical benefits mediated through shared visualisation and real-time instructional support. Nevertheless, the present findings remain limited to immediate procedural performance within a simulated educational environment. Further studies are required to determine whether these early advantages persist longitudinally, translate into supervised clinical competence, and inform the optimal sequencing of videolaryngoscopy and direct laryngoscopy within undergraduate airway management curricula.

The study results are consistent with the evidence synthesised in the Cochrane systematic review by Lewis et al. (17), which included multiple clinical trials in adult patients and demonstrated that video laryngoscopy significantly increases first attempt success compared to direct laryngoscopy. Although the magnitude of the effect observed in the study was larger, probably because the study population consisted of inexperienced operators, both studies support the view that improved glottic visualisation is a key mechanism underlying the superiority of videolaryngoscopy. In this regard, the randomised clinical trial by Silverberg et al. (18) in emergency intubation situations demonstrated that videolaryngoscopy was associated with higher success rates than direct laryngoscopy, especially when the procedures were performed by less experienced operators. This is particularly relevant to this study, as it reinforces the idea that videolaryngoscopy may reduce the technical difficulty encountered during initial training in airway management. The findings described by Griesdale et al. (19) also support the present findings. In their pilot clinical trial in critically ill patients, video laryngoscopy provided better visualisation of glottic structures than direct laryngoscopy. Although the difference in overall success rate was not as pronounced as in the study, the authors concluded that videolaryngoscopy may reduce the technical difficulty of the procedure, which is consistent with the observation in this study that fewer attempts were needed to achieve intubation. Similarly, the experimental study by Pieters et al. (20), performed on manikins with experienced and novice personnel, demonstrated that video laryngoscopes facilitate a faster learning curve than the conventional Macintosh laryngoscope (direct laryngoscopy). This is consistent with the present findings, where trainees using video laryngoscopy achieved significantly higher success rates from the first attempts.

The classic study by Malik et al. (21) evaluated different video laryngoscopy devices in patients with cervical immobilisation, a situation known to increase the difficulty of intubation. In that study, the video laryngoscopy devices showed clear advantages over the Macintosh laryngoscope in terms of glottic visualisation and ease of intubation. Although the clinical context differs from our work, these findings support the view that VL can reduce the technical complexity of the procedure in anatomically difficult situations or when mobility is limited. More recently, the meta-analysis by Gunning et al. (15) specifically evaluated the role of video laryngoscopy in teaching direct laryngoscopy skills. The authors concluded that the use of videolaryngoscopy during training enhances learning due to shared visualisation between instructor and trainee. This pedagogical feature is fully consistent with this study, in which participants reported greater perceived control of the procedure and lower stress levels when using video laryngoscopy. The multicentre VISI clinical trial by Garcia-Marcinkiewicz et al. (22) demonstrated that video laryngoscopy significantly increases first attempt intubation success in young infants. Although this study focused on a paediatric population, its results reinforce the broader evidence that indirect visualisation of the airway can improve technical performance in different clinical settings.

In the educational setting, Öcal’s recent study (14) assessed the learning curve of medical students during tracheal intubation training. The results showed that students using video laryngoscopy achieved higher success rates and a faster learning curve than those using direct laryngoscopy. These findings are very consistent with our own and further support the hypothesis that VL can facilitate early training in airway management. Keresztes et al. (23), in a simulation-based study performed during cardiopulmonary resuscitation, compared different video laryngoscopy devices with the Macintosh laryngoscope in novice operators. The authors found that video laryngoscopy devices were associated with higher success rates and shorter intubation time. These findings are particularly relevant to the present study, as they confirm that, even in complex simulated scenarios, videolaryngoscopy can improve the performance of inexperienced operators.

### 4.3 Educational and clinical implications

Overall, the available evidence is consistent with the present findings and suggests that the advantages of videolaryngoscopy may be especially evident in operators with no previous experience in airway management. The marked difference observed in this study suggests that VL may play a particularly important role during the early stages of learning, when technical coordination and anatomical orientation are not yet fully developed. In educational settings, shared visualisation of the airway between instructor and trainee allows for immediate feedback and may facilitate observational learning during simulation training. The findings should be interpreted within the specific context of undergraduate simulation-based education. The higher success rate observed with videolaryngoscopy reflects improved initial performance in a controlled high-fidelity simulation environment among novice learners. These results do not imply that students achieved independent clinical competence in endotracheal intubation, nor that airway instrumentation should be considered a universal procedural requirement for all medical graduates. Rather, videolaryngoscopy may provide a useful educational bridge during the early stages of airway management training by improving glottic visualisation, facilitating instructor feedback and reducing the technical barriers associated with direct laryngoscopy. Findings related to procedure-associated complications should be interpreted primarily as indicators of initial technical performance and procedural risk within a controlled simulation-based educational context rather than as estimates of actual clinical harm. In novice undergraduate learners, these measures provide relevant information regarding early-stage safety, technical precision and procedural development, whilst avoiding overinterpretation of simulation-derived outcomes as direct predictors of real-world patient injury.

### 4.4 Limitations

Although this approach ensures methodological consistency among participants, it may reduce the variability commonly found in real clinical settings, where airway difficulties and patient characteristics may differ substantially. Although the results of this study are promising, several limitations must be taken into account when interpreting the findings. The study was conducted in a high-fidelity clinical simulation environment. This approach ensured standardisation, safety and control of the educational intervention, but does not fully replicate the anatomical variability, physiological responses or emotional pressure associated with airway management in real patients. An additional limitation relates to the inherent constraints of the simulation model used. Although the Airway Larry® simulator allowed structured assessment of procedural safety and observable technical errors, it does not permit direct objective confirmation of injuries to non-visible distal airway structures, such as the distal trachea or pulmonary parenchyma. Consequently, recorded complications should be interpreted as indirect markers of technical risk and procedural safety rather than as actual clinical injury events. This limitation should be considered when interpreting complication-related findings and reinforces the need for cautious extrapolation of simulation-based safety outcomes to real-world clinical practice. Therefore, the results should be interpreted as evidence of competence acquisition at an early stage of training, and not as direct proof of clinical performance in real-life emergency situations. The study population consisted exclusively of fourth-year medical students with no prior experience in advanced airway management. This design is suitable for assessing the initial phase of learning, but limits the generalisation of the results to residents, doctors in specialist training or experienced practitioners. The marked advantage observed with video laryngoscopy may be particularly relevant for novice operators; therefore, future studies should analyse whether this effect persists across different levels of clinical experience. Some attitudinal variables, such as perceived stress and perceived time control, were based on the participant’s own assessment. Although these variables are relevant in simulation-based education, they may be influenced by individual psychological factors, prior exposure to simulation, or perceived self-efficacy. Future studies should combine these measures with validated stress scales, objective physiological markers and external assessment by trained observers. The study was conducted at a single academic institution. Although this increased internal consistency and allowed for a highly standardised educational intervention, multicentre studies are needed to confirm the external validity of the AIR-MASTER-SIM model across different medical schools, simulation centres and curricular contexts. A further limitation is that the study assessed immediate performance following simulation-based training and did not evaluate medium- or long-term retention of airway management competencies. Therefore, it remains unknown whether the differences observed between videolaryngoscopy and direct laryngoscopy would persist after several months or after repeated practice. In addition, the study was conducted in a simulated environment and did not assess the transfer of skills to supervised clinical practice. Future studies should include longitudinal follow-up, refresher training sessions and structured assessment of transfer to real clinical settings under appropriate supervision. This work did not assess the long-term retention of airway management skills or the transfer of skills acquired through simulation to supervised clinical practice. Future work should include longitudinal follow-up at 3, 6 and 12 months, analyse the effect of refresher sessions, and explore whether early exposure to video laryngoscopy in simulated settings subsequently improves clinical performance and patient safety outcomes. Based on the identified limitations, a research agenda structured around five priority areas is proposed. These areas enable the study’s limitations to be transformed into opportunities for methodological, educational and clinical advancement.

### 4.5 Proposed roadmap for future research

Building on the limitations identified above, the proposed roadmap for future research provides a structured framework to advance airway management education beyond the controlled simulation setting. Future investigations should prioritise external validation of the AIR-MASTER-SIM model across different institutions and educational environments, in order to determine whether the observed benefits of videolaryngoscopy are reproducible across heterogeneous learner populations and training contexts. A second priority should be the evaluation of how simulation-acquired competencies are consolidated over time and transferred into supervised clinical practice. This step is essential to determine whether early technical advantages observed in simulation translate into meaningful improvements in real-world procedural performance, clinical decision-making and patient safety. In parallel, future studies should compare different videolaryngoscopy devices and airway management platforms, taking into account not only procedural success, but also learning curve, usability, instructor feedback, accessibility and curricular feasibility. Finally, airway management training should progressively move toward competency-based educational models that integrate simulation, structured assessment, feedback and emerging technologies. Tools such as immersive simulation, artificial intelligence-assisted feedback and adaptive learning systems may help personalise training pathways and strengthen both technical and non-technical competencies. Overall, this roadmap aims to transform the findings of the present study into a broader research agenda focused on validation, transferability, technological refinement and curriculum integration.

## 5 Conclusion

Videolaryngoscopy was associated with markedly higher intubation success rates, fewer attempts, shorter procedure time and fewer complications than direct laryngoscopy when used by medical students with no prior experience in airway management in a high-fidelity simulation environment. Participants trained with videolaryngoscopy reported greater perceived control of procedure time and lower levels of stress during the simulated scenario. These findings suggest that videolaryngoscopy may be a particularly useful tool during the early stages of airway management training all under the methodological support of high-fidelity clinical simulation. These findings support the integration of videolaryngoscopy into simulation-based medical education programmes aimed at developing airway management competencies in novice operators.

## Data Availability

The original contributions presented in the study are included in the article/supplementary material, further inquiries can be directed to the corresponding author.
